# The Pathogenesis of Hydrocephalus Following Aneurysmal Subarachnoid Hemorrhage

**DOI:** 10.3390/ijms22095050

**Published:** 2021-05-10

**Authors:** Lu-Ting Kuo, Abel Po-Hao Huang

**Affiliations:** Division of Neurosurgery, Department of Surgery, National Taiwan University Hospital, Taipei 100, Taiwan; kuoluting@gmail.com

**Keywords:** cerebral aneurysm, subarachnoid hemorrhage, hydrocephalus, shunt, pathogenesis, inflammation

## Abstract

Hydrocephalus is a common complication of aneurysmal subarachnoid hemorrhage (aSAH) and reportedly contributes to poor neurological outcomes. In this review, we summarize the molecular and cellular mechanisms involved in the pathogenesis of hydrocephalus following aSAH and summarize its treatment strategies. Various mechanisms have been implicated for the development of chronic hydrocephalus following aSAH, including alterations in cerebral spinal fluid (CSF) dynamics, obstruction of the arachnoid granulations by blood products, and adhesions within the ventricular system. Regarding molecular mechanisms that cause chronic hydrocephalus following aSAH, we carried out an extensive review of animal studies and clinical trials about the transforming growth factor-β/SMAD signaling pathway, upregulation of tenascin-C, inflammation-dependent hypersecretion of CSF, systemic inflammatory response syndrome, and immune dysregulation. To identify the ideal treatment strategy, we discuss the predictive factors of shunt-dependent hydrocephalus between surgical clipping and endovascular coiling groups. The efficacy and safety of other surgical interventions including the endoscopic removal of an intraventricular hemorrhage, placement of an external ventricular drain, the use of intraventricular or cisternal fibrinolysis, and an endoscopic third ventriculostomy on shunt dependency following aSAH were also assessed. However, the optimal treatment is still controversial, and it necessitates further investigations. A better understanding of the pathogenesis of acute and chronic hydrocephalus following aSAH would facilitate the development of treatments and improve the outcome.

## 1. Introduction

Aneurysmal subarachnoid hemorrhage (aSAH) remains a devastating disease that is characterized by a high mortality rate and significant morbidity amongst survivors [1,2]. Hydrocephalus is a frequently encountered complication following aSAH and is classified as acute (0–3 days post-SAH), subacute (4–13 days post-SAH), or chronic (14 days post-SAH) [3,4]. Acute hydrocephalus necessitates the placement of an external ventricular drain (EVD) to reduce deleterious secondary effects after the aneurysm bleed, with up to 48% of EVD recipients requiring ventriculoperitoneal (VP) shunt insertion [5,6,7]. Chronic hydrocephalus has been reported in 9–64% of aSAH patients; placement of a shunt system improves clinical outcome in aSAH [8,9,10,11]. Early cerebrospinal fluid (CSF) drainage with an EVD reduces the content of blood-clotting products and protein in the CSF, which reduces the incidence of obstruction in the CSF flow pathway [12,13]. However, the prolonged use of an EVD may complicate the treatment of aSAH and increase the risks of meningitis and/or ventriculitis, which not only impacts the outcome but also influences the possibility of the patient becoming shunt-dependent [14,15]. Notably, complications relating to shunt placement are common, including intracerebral hemorrhage, shunt dysfunction, overdrainage, and infection [16,17].

Various mechanisms have been implicated as causative factors for the development of chronic hydrocephalus following aSAH, including alterations in CSF dynamics, obstruction of the arachnoid granulations by blood products, and adhesions within the ventricular system [18,19,20]. Prior studies evaluating factors associated with VP shunt placement have found that older age, female sex, a history of hypertension, high Fisher grade on the initial computed tomography (CT) scan, a low initial Glasgow Coma Scale (GCS) score, a higher Hunt and Hess grade at admission, amount of subarachnoid blood, presence of intraventricular hemorrhage (IVH), in-hospital complications (including pneumonia, meningitis, vasospasm, and ischemic stroke), a larger third ventricular diameter at admission, history of sympathomimetic drug use, hyponatremia, aneurysm location within the posterior circulation; prolonged EVD, aneurysm treatment modality, and aneurysm size were predictors of shunt-dependent hydrocephalus [5,21,22].

**Figure 1:**
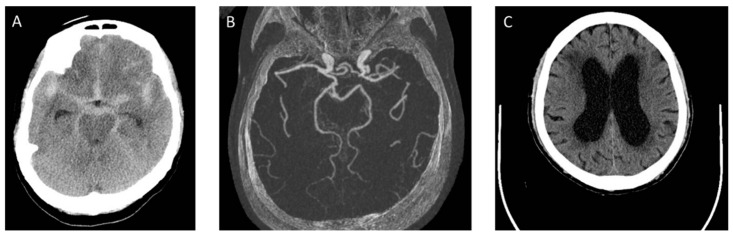
The series of computed tomography (CT) images of a case with aSAH. (**A**) A 68 years-old female patient presented with SAH at admission. There is no IVH and no acute hydrocephalus; (**B**) a ruptured left posterior communicating artery aneurysm was identified and coiled; (**C**) on 12 days after aSAH, CT showed hydrocephalus. She developed progressive ataxia and cognitive dysfunction.

Chronic hydrocephalus following aSAH reportedly contributes to poor neurological outcomes and severe cognitive deficits [22,23,24,25], which was also found in our cases (Figure 1). The development of predictive models that could stratify patients with aSAH based on their risk of developing shunt-dependent chronic hydrocephalus is important. Data from these models could provide guidance for neurosurgeons as to the earlier replacement of an EVD with a shunt system for higher-risk patients, with the associated benefits of a lower incidence of EVD infection, shortened hospital stays, lower treatment costs, and improved functional outcomes. In contrast, patients at lower risk of shunt dependency could undergo a more aggressive EVD weaning protocol. Very few such scoring systems have been described in the literature [8,26,27].

Following aSAH, evidence suggests that extensive fibrosis in the subarachnoid space may be an important cause of chronic hydrocephalus development [18,28]. A rapid inflammatory cell response occurs in the leptomeninges following aSAH, with polymorphonuclear cells dominating during the first 24 h and mononuclear cells thereafter [29,30]. These inflammatory cells secrete cytokines that trigger a fibroproliferative reaction by acting as mitogens and chemoattractants for fibroblasts. Reportedly, inflammatory cytokines and growth factors such as tumor necrosis factor (TNF)-α, interleukin (IL)-1, IL-6, platelet-derived growth factor (PDGF), and transforming growth factor (TGF)-β are upregulated in the acute stage of aSAH [31,32,33]. Thrombin is also released by the blood-clotting cascade in the CSF of patients with aSAH [34,35]. Thrombin, TGF-β, and PDGF reportedly promote human leptomeningeal cell proliferation in culture, and hydrocephalus develops in mice injected with intracerebral TGF-β [36].

## 2. TGF and Its Antagonists

Post-hemorrhagic blood-clotting products with fibrosis of the leptomeninges and arachnoid granulations may reduce the circulation of CSF, suppress CSF absorption, and reduce drainage, leading to the development of hydrocephalus [37,38]. The three mammalian isoforms of TGF-β (TGF-β1, TGF-β2, and TGF-β3) are small secreted homodimeric signaling proteins [39]. They coordinate and control various cellular processes, including cell proliferation and differentiation, apoptosis, migration, wound healing, angiogenesis, immune cell function, maintenance of the extracellular matrix, and other functions in many different cell types [39,40,41]. In the central nervous system (CNS), all three isoforms are produced by both glial and neuronal cells [42,43]. The TGF-β family can mediate signaling by binding to two serine threonine kinase receptors on the cell surface, TGF-β type-1 and type-2 receptors, inducing the phosphorylation and activation of Smad 2/3 and Smad 4 transcription factors [44,45], initiating multiple intracellular signaling, and exerting profibrotic effects [46,47]. The TGF-β1 isoform is the most abundant cytokine in the CNS and is recognized as having a crucial role in brain injury and regulation of CNS development [48]. TGF-β1 can also activate multiple downstream intracellular signaling pathways to exert diverse cellular effects, including the Rho/Rho-associated coiled-coil-forming protein kinase (Rock) pathway, protein kinase C (PKC)-δ pathways, and Ras/mitogen-activated protein kinase/Erk1/2 pathways [49,50,51]. The fibrotic response of various organs is a highly complex and multifaceted process. The TGF-β1/Smad/connective tissue growth factor (CTGF) pathway is involved in the pathogenesis of various fibrotic diseases [47,52]. TGF-β1 levels have been reported to be higher in the CSF following aSAH, especially in patients with hydrocephalus, which implies its role in the pathogenesis of subarachnoid fibrosis and chronic hydrocephalus following aSAH [53,54,55]. As a major downstream mediator of TGF-β1/Smad signaling in many cell types, CTGF is regarded as an important amplifier of the pro-fibrogenic action of TGF-β1 in a variety of tissues [56,57]. CTGF expression is also significantly increased following aSAH [58,59]. In experimental aSAH models, TGF-β1 concentrations in CSF and protein TGF-β1 levels are significantly increased in periventricular brain tissues [60,61]. Immunofluorescent studies have revealed that glial cells are mainly responsible for the expression of TGF-β1 in parenchyma following aSAH, especially in the subependymal areas [59,62]. This research has described a two-peak response of TGF-β1 in the CSF [59,62]. The first TGF-β1 peak is basically exogenous and derived from the excess storage of TGF-β1 in platelets, which can be released by platelet degranulation following aSAH [59,62]. The second TGF-β1 peak is attributed to endogenous sources, whereby TGF-β1 acts as a chemoattractant for inflammatory cells and platelets and interacts with other cytokines that further promote the local production of TGF-β1 in the CSF and choroid plexus [59].

Some TGF-β1 antagonists and TGF-β1 signaling pathway inhibitors alleviate chronic hydrocephalus and improve behavioral outcomes in rat aSAH models [58,59]. Decorin, a member of the small leucine-rich extracellular matrix proteoglycans, has exhibited significant inhibitory effects on the TGF-β1/Smad/CTGF axis, protecting against extracellular matrix accumulation with antifibrotic effects [59,63]. Decorin acts as a natural antagonist to TGF-β by forming complexes with TGF-β to neutralize and suppress its function. Decorin also impedes activation of TGF-β receptors and inhibits downstream signaling pathways by competitive inhibition [64,65]. Several studies have demonstrated the therapeutic role of decorin in suppressing the fibrogenic response in a wide variety of tissues and organs, including the brain and spinal cord after injury [59,63,66,67]. In animal studies, decorin attenuates the formation of glial scarring in the CNS, reduces epidural fibrosis in rats following laminectomy, and effectively suppresses the development of post-hemorrhagic chronic hydrocephalus [59,67,68]. As a small molecular peptide and competitive antagonist for TGF-β1, this LSKL peptide can easily cross the blood-brain barrier and protect against subarachnoid fibrosis and chronic hydrocephalus following aSAH, as shown by a rat model of aSAH [69].

## 3. Tenascin-C

The expression of tenascin-C (TNC), a matricellular protein, is extremely low in adult tissues under normal physiological conditions. TNC regulates cellular phenotype and promotes the migration and proliferation of myofibroblasts, as well as neuroinflammatory cascades [70,71,72]. TNC is upregulated by various pro and anti-inflammatory cytokines and interleukins, including TNF-α, IL-1, PDGF, and TGF-β [73,74,75]. Upregulation of TNC in the serum and CSF is associated with worse neurological grades at admission, a greater amount of hemorrhage on CT scan, and symptomatic vasospasm following aSAH [76,77]. TNC may activate the proliferation of leptomeningeal cells and promote tissue fibrosis by increasing the synthesis of type I and III collagen [70,77]. In preclinical studies, the involvement of TNC in neuronal apoptosis and blood-brain barrier disruption following aSAH reportedly occurs via the activation of mitogen-activated protein kinases and nuclear factor-kappa B [78,79,80]. TNC may be involved in blood-brain barrier disruption, neuronal apoptosis, and cerebral vasospasm following aSAH [76,77]. Thus, TNC may cause leptomeningeal collagen synthesis and fibrosis, as well as brain injuries with decreased brain parenchymal volume contributing to subsequent ventricular enlargement, resulting in the development of chronic hydrocephalus, which is consistent with findings from a CSF study in humans [81,82]. The induction of leptomeningeal collagen synthesis within the first 48 to 72 h following aSAH is consistent with the highest increase of CSF TNC concentrations occurring in the first 3 days following aSAH [82,83].

## 4. SIRS Following aSAH

SAH causes a systemic inflammatory response syndrome (SIRS) that involves complex interactions amongst immune cells, inflammation, coagulation, sympathoadrenal activation, endothelial cell activation, and dysfunction [84]. This complex process leads to a procoagulant reaction, tissue hypoperfusion, microthrombosis, and compromised microcirculation, which ultimately leads to multiorgan failure [84,85,86]. Release of catecholamines into the systemic circulation following aSAH may cause arrhythmias and neurogenic pulmonary edema. Clinically, SIRS has been defined by the presence of two or more of the following: a temperature <36 or >38 °C, a heart rate >90 bpm, a respiratory rate of >20 breaths/min, and a white blood cell count of <4000 or >12,000/mm^3^ [84,87]. This response produces high levels of circulating cytokines, such as IL-1, IL-6, and TNF-α, which are key mediators of systemic inflammation [88]. The clinical manifestation includes fever, leukocytosis, tachycardia, and tachypnea. It has been reported that SIRS is present at admission in over half of the patients and 63–85% of patients within 4 days following aSAH [89,90]. SIRS is associated with worse Hunt and Hess grades and larger amounts of aSAH [87]. SIRS is also an independent predictor of angiographic vasospasm, systemic complications, unfavorable outcomes, and death [90]. Interestingly, SIRS scores were found to be significantly higher with clipping than with coil embolization in a cohort of patients with good-grade aSAH [91].

Inflammatory biomarkers are associated with the occurrence of vasospasm, delayed cerebral ischemia, and unfavorable outcomes [84]. In clinical studies, anti-inflammatory agents such as acetylsalicylic acid, NSAIDs, thromboxane synthase inhibitors, steroids, nitric oxide donors, and immunosuppressants have not been shown to be beneficial [84]. It is worth noting that there have been no well-designed or well-controlled clinical trials in this field. Thus, there is no approved intervention as of yet for treating neuroinflammation following aSAH. Interestingly, it has been shown that using a multimodal monitoring approach can potentially aid the development of therapeutics targeting different aspects of the inflammatory cascade following aSAH [85]. Last but not least, it would be interesting to look at the effect of anti-inflammatory agents on shunt dependency.

## 5. Immune Dysregulation Following aSAH

The immune response following aSAH has been described in recent publications. Following aSAH, systemic IL-6 levels increase rapidly, whereas IL-10 levels are reduced [92]. Neutrophils are increased both in the brain and in the blood, reflecting local and peripheral inflammation following aSAH [93]. Higher levels of intracerebral proinflammatory monocytes are found within 24 h than after 1 week [92]. Studies in mouse models of aSAH have revealed increased astrocyte and microglial activity, as well as severe motor deficits, which were associated with an increase in the percentage of caspase-3-positive apoptotic neurons [92]. An analysis of gene expression profiles in human peripheral blood samples indicates that the lymphocyte response is depressed and monocyte activity is enhanced following aSAH [94]. aSAH induces an early intracerebral infiltration and peripheral activation of innate immune cells [92,95]. Furthermore, microglia and astrocytic activation are present one week following aSAH [92]. Essentially, aSAH leads to SIRS and immune cells represent potential early and upstream therapeutic targets.

In addition to stroke-related SIRS, immune dysregulation plays an important role in brain injury and recovery. For example, the spleen contracts following ischemic stroke, activating a peripheral immune response that may exacerbate ongoing brain injury [96]. Analyses of the neutrophil-to-lymphocyte and platelet-to-lymphocyte ratios reveal an early state following aSAH [94,97]. While there is growing data suggesting that peripheral immune dysregulation following hemorrhagic strokes may be important in brain injury pathogenesis and outcomes, details of the crosstalk between the brain and the immune system remain unclear [98].

## 6. Inflammation-Dependent Hypersecretion of CSF Following aSAH

**Figure 2:**
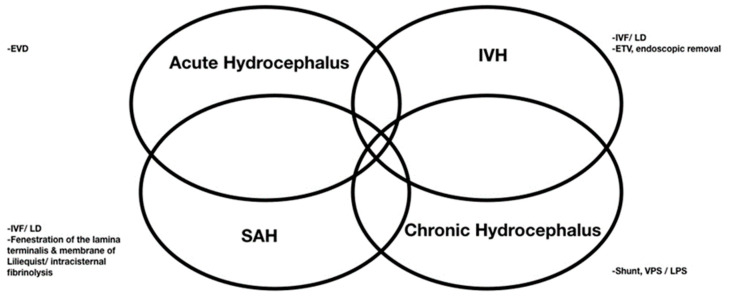
Surgeries applied for four mechanisms underlying the pathophysiology of aSAH-induced brain injury.

The rate of shunt dependency after treated aSAH ranges from 17.2% to 31.2% [25,27]. The four mechanisms underlying the pathophysiology of aSAH-induced brain injury are acute obstructive hydrocephalus, a mass effect exerted by IVH, SAH-related cytotoxic blood degradation products in the adjacent brain tissue, and chronic hydrocephalus [99] (Figure 2). Acute hydrocephalus requires EVD management, while using EVD may also cause chronic hydrocephalus. When chronic hydrocephalus progresses, EVD cannot be removed and may need a permanent shunt. The clearance of blood clots from the ventricles has therefore become a major therapeutic goal. The modified Graeb scale, the qualitative measurement of IVH and acute hydrocephalus, is reportedly the simplest model that correlates well with shunt dependency following aSAH; a modified Graeb score higher than 12 identifies patients at risk with high specificity (85%) [100]. EVD has been recommended for IVH cases with acute hydrocephalus but is characterized by frequent clot obstruction and infection risks associated with prolonged drainage [101]. In addition, EVD replacement may affect the risk of shunt dependency; larger volumes of CSF drainage per day and prolonged EVD are both associated with shunt dependency [102,103,104,105]. Further studies are required to develop an evidence-based EVD management strategy that minimizes the risk of shunt dependency.

The use of intraventricular fibrinolysis (IVF), such as recombinant tissue plasminogen activator (rtPA) or urokinase, was introduced to overcome the above problems with EVD and has been shown to be beneficial in selected cases. In the recent CLEAR III study, the comparison of postoperative outcomes between alteplase and saline irrigation in the thrombotic removal of IVH found that although alteplase was associated with lower mortality rates, there was no substantial improvement in functional outcomes, because most of the survivors were severely disabled [106]. Despite the AHA/ASA Guidelines claiming that the efficacy of endoscopic surgery for IVH is uncertain, its efficacy has been reported in several studies. A meta-analysis of randomized controlled trials (RCTs) and observational trials published between 1970 and 2013 showed that applying the endoscopic approach with EVD led to less shunt dependency and a shorter length of ICU stay compared with the IVF approach [107]. In another couple of studies, Longatti et al. and Chen et al. performed endoscopic IVH evacuations and found that the endoscopic approach effectively reduced shunt dependency and had more favorable outcomes [108,109]. In the study by Oertel et al. involving 34 patients who underwent endoscopic third ventriculostomy (ETV) for obstructive hydrocephalus due to IVH, ETV was a safe treatment option with less risk of infection and less shunt dependency compared with EVD [110]. A recently conducted randomized study and individual patient data meta-analysis indicate that the combination of IVF plus lumbar drainage for IVH significantly reduced shunt dependency compared with IVF alone [111].

However, most of these studies focused on primary ICH and IVH patients, so the generalization of aSAH patients with IVH may not be appropriate. A randomized trial on IVF in IVH secondary to aSAH is mandatory. A phase III, open-label RCT, FIVHeMA, was recently initiated to compare IVF plus EVD with the standard of care (i.e., EVD alone) in aSAH [112]. The plan is to include 440 patients for demonstrating a 10% increase in the rate of good functional outcomes in the EVD plus IVF group compared with the EVD-alone group. To obtain such a sample, a multicenter trial is required. To date, 17 research sites in France have agreed to participate. A previous study has shown that in endovascular-treated aSAH patients, IVF neither reduced permanent shunt dependency nor influenced functional outcomes [113]. Despite the established effects of IVF on IVH resolution, it appears less effective in aSAH compared with ICH. In particular, the radiological permeation of CSF pathways does not always exclude the need for shunting for aSAH patients with IVH. It has been shown that lumbar drainage after radiological permeation of the ventricular system (especially the third and fourth ventricles) is associated with a decrease in shunt dependency, and this promising strategy is now the subject of a randomized trial [111,114]. It has also been shown that intracisternal fibrinolysis reduces the incidence of hematoma in the basal cisterns at 48 h following aSAH, with significant, accompanying reductions in the proportions of patients with poor neurological outcomes and those who are shunt-dependent [115].

Despite the fact that intraventricular blood clots are dissolved, blood derivatives enter the parenchyma and may adversely affect functional structures of the brain; smaller blood clots may obstruct the perivascular (Virchow–Robin) space and subsequently the glymphatic system, with detrimental consequences for CSF/interstitial fluid (ISF) flow. These clots, as well as blood cells and blood derivatives in the perivascular space, destabilize the blood-brain barrier from the brain parenchyma side and functionally weaken the neurovascular unit. This may lead to further accommodation of serum proteins in the ISF, particularly in the perivascular space, further contributing to the adverse effects on the neuronal microenvironment. Finally, the arterial (Pacchionian) granulations have to cope with ISF containing this “blood, cell, and protein cocktail”, resulting in the obstruction and insufficient function of the arterial granulations, followed by a malresorptive hydrocephalus. In light of greater knowledge about the physiologic and pathophysiologic clearance of CSF and ISF, critical discussions and reevaluation of our current therapeutic strategies for treating IVH are needed if we are to successfully treat patients suffering from this severe type of stroke [116].

## 7. Endoscopic IVH Removal

Endoscopic IVH removal can be useful in certain cases. Although the published series on endoscopic IVH removal detail results similar to those with IVF, no randomized trial has proven the superiority of this approach over EVD + IVF or EVD alone. Although the combination of coiling and endoscopic IVH removal in aSAH patients has proven safe and effective, given the technical demand of endoscopic IVH removal, evidence demonstrating its superiority over EVD + IVF is needed before it can be widely adopted [117,118,119,120]. This can be performed in the hybrid room with a multidisciplinary team with coiling performed before or after endoscopic IVH removal [119,120]. The hybrid operating room enables the two treatment approaches to be performed without the need to transfer the patient, and thereby minimizes the transition time between the modalities. In addition, intraoperative cone-beam CT can be used to confirm adequate decompression and document the volume of residual IVH (and may determine the need for additional IVF) [120]. Another concern is the occurrence of EVD-related hemorrhagic complications in endovascular-treated patients. Most of the recent literature supports the safety of EVD placement in the peri-endovascular treatment period [121]. In a recent meta-analysis of 13 studies evaluating 516 patients with antiplatelet therapy, and 647 patients without antiplatelet therapy, patients receiving ventriculostomy and antiplatelet therapy during endovascular treatment of acutely ruptured intracranial aneurysms increases the risk of EVD-related hemorrhages (20.9% vs. 9%), although most of them are small and asymptomatic [122]. When EVD is performed before endovascular procedures requiring antiplatelet therapy, the hemorrhagic risk is minimized [122]. A recent paper has supported the contention that pre-embolization EVD does not increase hemorrhagic complications and is associated with better functional outcomes at discharge [123]. In that study, compared with dual therapy, single antiplatelet therapy was associated with a lower rate of major bleeding (7% vs. 1.7%) [122].

## 8. Coil vs. Clip and Their Relationship with Shunt Dependency

Although the influence of treatment modality (surgical clipping versus endovascular coiling) on shunt dependency remains controversial, it has been postulated that open surgery allows irrigation and removal of subarachnoid clots and thereby reduces the probability of chronic hydrocephalus [9,124]. Some studies have suggested that endovascular treatment is independently associated with the development of chronic hydrocephalus in aSAH patients [124,125]. However, other research has demonstrated a significantly lower incidence of chronic hydrocephalus after endovascular treatment compared with after surgical clipping [126]. Surgical clipping via the traditional pterional transsylvian approach with arachnoid membrane dissection may directly alter CSF flow dynamics and resorption [126]. In a retrospective review of 839 patients with aSAH [127], endovascular coiling was associated with a lower risk of shunt dependency for Fisher grade 2 patients (2% vs. 13% with clipping, *p* = 0.043), while surgical clipping lowered the likelihood of shunt dependency in patients with Fisher grade 4 aSAH (23% vs. 44%, *p* = 0.004). In another retrospective study including 1448 aSAH patients, microsurgical clipping conferred a two-fold higher risk of chronic hydrocephalus over coiling alone [128]. Other studies reported no significant difference for the predictive risks of shunt-dependent hydrocephalus between surgical clipping and coiling groups [24,129,130,131,132]. Also, a multicenter aSAH database in Japan consisting of 566 patients treated for aSAH has revealed that shunt dependency following aSAH occurred significantly more frequently in patients who underwent clipping than in those treated with endovascular coiling (30% vs. 16%) [126]. Last but not least, another large database of 10,899 aSAH patients which consisted of 6593 patients receiving clipping and 4306 receiving coiling reported that their incidence of VP shunt insertion was similar (9.3% vs. 10.5%) between the surgeries [133]. Nevertheless, these complicated data are difficult to interpret. The risk of shunt-dependent hydrocephalus is significantly different between surgery groups because the previous publications cannot provide the key factors to determine the need for shunt treatment and the determinant of EVD weaning, resulting in the variability for what may be perceived as a shunt-insertion threshold.

Some surgical techniques have been shown to decrease the risk of shunt dependency. Tandem fenestration of the lamina terminalis and membrane of Liliequist has been shown to decrease shunt dependency following surgical clipping or bypass following aSAH (17.9% vs. 3.2%) [11]. In addition, intracisternal fibrinolysis has been shown to decrease shunt dependency and improve functional outcomes following aSAH [115]. Once again, as these techniques are usually not quantified in studies, further analysis and study may be necessary to verify the therapeutic effects. The implication may be that an aneurysm should be treated as dictated by the neurovascular team, either endovascularly or microscopically. Treatment for IVH, SAH, and hydrocephalus (e.g., a shunt, IVF, and intracisternal fibrinolysis) may be considered separately. However, when microsurgical clipping is chosen, removal of IVH, the use of tandem fenestration of the lamina terminalis and membrane of Liliequist, and the use of intracisternal fibrinolysis are considered in the hope of decreasing subsequent shunt dependency.

## 9. Conclusions

To sum up, the development of shunt-dependent hydrocephalus following aSAH is multifactorial. The involvement of multiple cellular signaling pathways and inflammatory responses all contribute to its pathogenesis. This review integrates the updated knowledge about the clinical, molecular, prognostic, and therapeutic aspects of chronic hydrocephalus following aSAH. Recent literature has shown that prolonged use of EVD in the acute stage may lead to subsequent hydrocephalus. There is a need to standardize the EVD weaning process to decrease shunt dependency. Although lumbar drainage and IVF may decrease shunt dependency, those still need further validation. There have been no ideal treatment approaches for this devastating disease which points to a need for therapeutic strategies to target these underlying mechanisms. Understanding the risk factors and molecular pathophysiology related to the development of hydrocephalus following aSAH may help neurosurgeons to determine the intervention targeting these factors.

## Data Availability

Not applicable.
